# Whole-brain radiation therapy for brain metastases: detrimental or beneficial?

**DOI:** 10.1186/s13014-015-0466-9

**Published:** 2015-07-28

**Authors:** Cengiz Gemici, Gokhan Yaprak

**Affiliations:** Department of Radiation Oncology, Dr. Lutfi Kirdar Kartal Education and Research Hospital, Cevizli, Istanbul, Turkey

**Keywords:** Brain metastases, Whole-brain radiation therapy, Stereotactic radiosurgery

## Abstract

Stereotactic radiosurgery is frequently used, either alone or together with whole-brain radiation therapy to treat brain metastases from solid tumors. Certain experts and radiation oncology groups have proposed replacing whole-brain radiation therapy with stereotactic radiosurgery alone for the management of brain metastases. Although randomized trials have favored adding whole-brain radiation therapy to stereotactic radiosurgery for most end points, a recent meta-analysis demonstrated a survival disadvantage for patients treated with whole-brain radiation therapy and stereotactic radiosurgery compared with patients treated with stereotactic radiosurgery alone. However the apparent detrimental effect of adding whole-brain radiation therapy to stereotactic radiosurgery reported in this meta-analysis may be the result of inhomogeneous distribution of the patients with respect to tumor histologies, molecular histologic subtypes, and extracranial tumor stages between the groups rather than a real effect. Unfortunately, soon after this meta-analysis was published, even as an abstract, use of whole-brain radiation therapy in managing brain metastases has become controversial among radiation oncologists. The American Society of Radiation Oncology recently recommended, in their “Choose Wisely” campaign, against routinely adding whole-brain radiation therapy to stereotactic radiosurgery to treat brain metastases. However, this situation creates conflict for radiation oncologists who believe that there are enough high level of evidence for the effectiveness of whole-brain radiation therapy in the treatment of brain metastases.

## Background

For patients with brain metastases, stereotactic radiosurgery (SRS) has become an increasingly available treatment, especially as the range of stereotactic treatment technologies progresses. Although whole-brain radiation therapy (WBRT) has been used to treat brain metastases together with SRS, a recent trend promotes the use of SRS-alone. This trend is mainly based on the adverse effect of WBRT on neurocognitive functioning and quality of life scores. However, the data in this area is controversial and may not be reliable due to early evaluation of these end points after WBRT with a limited number of patients [1–4].

## Main text

In particular, the recent meta-analysis of Sahgal et al. [5, 6] has dissuaded many radiation oncologists against routinely adding WBRT to SRS for patients with 1 to 4 brain metastases, and have concluded that such addition is detrimental with respect to survival, especially for patients younger than 50 years of age. Sahgal et al. undervalue the role of WBRT, and think that “The sun is setting on WBRT” and “SRS alone is rising to be the standard of care” [6]. They base these conclusions on individual patient data meta-analysis of 3 randomized trials comprising only 364 patients. The patients in the meta-analysis are so heterogeneous with respect to tumor histologies, extracranial tumor stages, and systemic treatments that, brain directed treatments (SRS, WBRT) cannot determine the survival of the patients on their own. Unfortunately the distribution of the patients by age, recursive partitioning analysis (RPA), and number of brain metastases cannot equalize these heterogeneities between the groups. Although RPA classification is the most common method of determining patients with similar prognoses and survival outcomes, it is not the perfect method and moreover, the best way to classify these patients by prognosis and treatment outcome is the subject of ongoing studies [7, 8].

The primary endpoints in the randomized trials included in this meta-analysis were inconsistent, and none of them were designed for survival outcome [5]. Furthermore, the survival advantage found for the younger patients treated with SRS without WBRT in this meta-analysis may be the result of imbalances between the treatment groups rather than any real detrimental effect of WBRT. Patients were observed to be unequally distributed between the SRS, and WBRT + SRS arms with respect to tumor histologies. Recent studies demonstrated the prognostic value of tumor histology in patients presenting with brain metastases [9, 10]. Prognosis changes not only by tumor histology, but also with molecular histologic subtypes even within the same tumor histology [10]. Qi Shen et al. demonstrated that among breast cancer patients with brain metastases, the patients with Her2 expression had better survival compared to the patients with other molecular histologic subtypes [10].

Despite all the arguments against the addition of WBRT to SRS, this undervalued, and old-fashioned treatment modality provided higher progression-free survival rates (PFS), lower neurologic death rates (intracranial failure as a component of cause of death), lower intracranial relapse rates (both at initial sites of metastases and new intracranial sites), lower salvage cranial treatment rates in the randomized trials, and all these endpoints were statistically significant [3, 11–13]. What else can a radiation oncologist expect from WBRT? PFS is an important endpoint in oncology trials, and many new drugs, especially targeted therapies in medical oncology, have been approved only for this endpoint. Improved PFS obtained by WBRT was not considered as an important endpoint in one of the randomized trials of this meta-analysis, and WBRT addition to SRS was considered detrimental for health related quality of life in the same trial. However, the reality was not as the authors of this trial reported, and the defects found in this trial were expressed in a letter to the editors [3, 4].

The age limit of 50 years was thought to provide a survival advantage in the meta-analysis. However, it actually created two unequal groups with respect to tumor histologies. For example, among patients with lung cancer, which has rather poor survival, those younger than 50 years of age made up 57 % of the WBRT + SRS group, but only 29 % of the SRS group. Renal cell cancer cases were included only in SRS group younger than 50 years of age (16 %), but not in the WBRT + SRS group in the same age range (0 %). SRS may have immune stimulating effects for tumors with immunologic features, such as renal cell carcinomas and malignant melanomas, and may be more effective due to the intrinsic sensitivity of these tumors to high-fraction doses [14]. Beside these inequalities, molecular subtypes of breast cancer patients within the groups (ER+, triple-negative, Her2+) are not known either. Almost half of the patients between the groups differ histologically. The defects in the meta-analysis were expressed by a letter to the editors [15]. Although Sahgal et al. acknowledge that further research and prospective data are required because their findings are based on subgroup analysis and are hypothesis generating, they presented the data to the world as if the survival advantage observed in patients younger than 50 years of age was the result of treatment of brain metastases with SRS-alone, and the avoidance of detrimental effects of WBRT addition with respect to health related quality of life, and neurocognitive functions [5, 6, 16]. They base their assumptions only on the results of 68 patients out of 364 patients (less than 20 % of the whole group included in the meta-analysis), and additionally half of the patients out of these 68 patients were different from each other with respect to histology and prognosis. Although their meta-analysis could not analyze the toxicity aspects of SRS and WBRT, they considered the neurocognitive decline as an argument against WBRT addition to SRS in the management of patients with limited number of brain metastases [5, 6, 16].

While there are so many different prognostic variables related to the tumor histology or even within the same histologic tumor type, the comparison of the groups with respect to age alone can create a bias, and can lead to comparison of apples with oranges. The best method to equalize and balance the unknown factors related to the tumor itself is to perform a randomized study in patients with same tumor histology and molecular histologic subtype, same stage and systemic treatment for the extra cranial tumor. Although this is the best way to demonstrate the beneficial or detrimental effect of WBRT, we don’t think that this proposed trial will be able to be completed, knowing the fact that even the three nationally funded randomized trials could enroll only 364 patients.

Concerns about the neurocognitive effects of WBRT have led to several approaches that mitigate cognitive dysfunction, such as pharmacologic interventions (ex. memantine) and hippocampal sparing during WBRT [17, 18]. Transferring these findings to the clinic can preserve the quality of life, and neurocognitive functions of the patients who receive WBRT.

An argument against adding WBRT to SRS is that by withholding WBRT, it can be later used in cases of relapse as salvage treatment, and this approach may prevent the adverse effects of WBRT in patients who would not need it. In the meta-analysis at least 50 % of the patients initially treated with SRS-alone needed salvage treatment [5, 16]. However, to allow patients to relapse while offering close follow-up, and then to provide salvage treatment is not a correct oncologic approach, especially when the cure is the goal. Patients with small-cell lung cancer treated with prophylactic cranial irradiation, and patients with solid tumors presenting with single brain metastases are good examples. Close follow-up after treatment with SRS-alone, not only induces fear of recurrence in the patients, but also may not be cost-effective.

## Conclusion

Before considering WBRT as detrimental to patients with brain metastases, we should question the validity of the data in the recent meta-analysis. There is sufficient high-level evidence for the effectiveness of WBRT. In fact what should be investigated in the management of brain metastases, should be the efficacy and the toxicity of radiation dose and fraction used frequently during WBRT (30Gy/3Gy fractions), and other techniques like hippocampal sparing, and pharmacologic interventions to reduce the potential long-term toxicities of WBRT.

The recent meta-analysis may unduly influence oncologists world-wide, and has even influenced the American Society for Radiation Oncology when initially presented at their annual meeting in 2013, leading to a wide-spread campaign against routine use of WBRT in the management of brain metastases [5, 6]. This may create legal problems in near future for oncologists who, with a high level of evidence, consider WBRT to be beneficial for their patients.

## Consent

Written informed consent was obtained from the patient for the publication of this report and any accompanying images.
